# A Comparison of Risperidone and Buspirone for Treatment of Behavior Disorders in Children with Phenylketonuria

**Published:** 2014

**Authors:** Afshin FAYYAZI, Elham SALARI, Ali KHAJEH, Abdi GAJARPOUR

**Affiliations:** 1 Department of Pediatric Neurology, Hamadan University of Medical Sciences, Hamadan, Iran; 2 Child & Adolescent Psychiatrist, Hamadan University of Medical Sciences, Hamadan, Iran; 3 Department of Pediatric Neurology, Zahedan University of Medical Sciences, Zahedan, Iran; 4 Hamadan University of Medical Sciences,Hamadan, Iran

**Keywords:** Behavior disorders, Aggression, Phenylketonuria, Risperidone, Buspirone

## Abstract

**Objective:**

Many patients with late-diagnosed phenylketonuria (PKU) suffer from severe behavior problems. This study compares the effects of buspirone and risperidone on reducing behavior disorders in these patients.

**Materials & Methods:**

In this crossover clinical trial study, patients with severe behavior disorders after medical examination were randomly divided into two groups of two 8-week crossover treatments with risperidone or buspirone. Patient behavioral disorders before and after treatment by each drug was rated by parents on the Nisonger Child Behavior Rating Form (NCBRF), and after treatment by each drug, were assessed by a physician through clinical global impression (CGI).

**Results:**

Thirteen patients were able to complete the therapy period with these two medications.

The most common psychiatric diagnoses were intellectual disability accompanied by pervasive developmental disorder NOS, and intellectual disability accompanied by autistic disorder. Risperidone was significantly effective in reducing the NCBRF subscales of hyperactivity disruptive/stereotypic, and conduct problems. Treatment by buspirone only significantly decreased the severity of hyperactivity, but other behavior aspects showed no significant differences. Assessment of the severity of behavior disorder after treatment by risperidone and buspirone showed significant differences in reducing hyperactivity and masochistic/stereotype.

**Conclusion:**

Although buspirone is effective in controlling hyperactivity in patients with PKU, it has no preference over risperidone. Therefore, it is recommended as an alternative to risperidone.

## Introduction

Children with late-diagnosed phenylketonuria (PKU) suffer from severe behavioral problems except for intellectual disability. Hyperphenylalaninemia causes several behavioral disorders through suppression of dopamine and serotonin turnover that affects the prefrontal cortex of the brain (1). Common behavior problems in these children include autistic behaviors, aggression, hyperactivity, impulsive behavior, attention-deficit, and disruptive stereotypic movements (1,2). Lack of a pervasive PKU screening programs in Iran until one year ago have led to encountering a high number of late-diagnosed PKU patients with severe behavioral disorders. Parents of these children usually complain of uncontrollable and annoying behavior of their children, in addition to difficult feeding with phenylalanine-restricted diet, and they are often looking for a treatment to reduce behavioral problems. Current treatments available for reducing these types of disorders include antipsychotics and mood stabilizers, receptor 2α agonists, tricyclic antidepressants, and stimulant drugs such as Ritalin (3,4).

Several studies have shown the effectiveness of risperidone, atypical antipsychotics, and antagonists of dopamine D2 and serotonin 5-HT 2A receptors in controlling behavior problems (4), pervasive developmental disorders (5-10), disruptive behavior disorders (11-14), and intellectual disability (15-18). 

Hence, this drug has become the first-choice treatment for most behavioral disorders in patients with brain structural disorders. One of the major problems in the management of behavior disorders by antipsychotic drugs in these patients is the lack of clinical response and the occurrence of adverse effects in a portion of patients. Therefore, in these patients, alternative drugs are needed.

Some studies have reported the effectiveness of buspirone, a partial agonist of the 5-hydroxytryptamine- 1A (5-HT 1A) in the control of hostility and aggressive behaviors secondary to brain structural disorders (19). 

Another study has shown the effects of buspirone on the control of behavior disorders caused by brain trauma (20). In addition, some reports have indicated the efficacy of this drug in reducing anxiety and aggression in children with intellectual disabilities and autistic disorders (21,22).

The main purpose of this study compares the effects of risperidone and buspirone for treatment of behavioral disorders in children with PKU. If our findings are confirmed, buspirone can be introduced as an alternative drug to this group. The reason of selection of PKU patients for comparison of these two drugs were: firstly, the needs of these patients for an alternative drug and lack of similar studies on these patients; and secondly, in studies on patients with different underlying brain problems require a detailed interpretation of the results was not possible.

## Materials& Methods

In this study, 42 patients aged 2–6 years with PKU (serum phenylalanine level > 6 mg/dl at the time of diagnosis, normal serum tyrosine) underwent initial assessment in the PKU specialized clinic of Besat Hospital of Hamadan, which is a provincial referral center for PKU patients. Then, cases with behavioral disorders (from the view of the parent and physical examination) were selected and referred to the Pediatric Psychiatry Clinic. 

A pediatric psychiatrist made the psychiatric diagnosis through a clinical interview based on DSM-IV-TR criteria for children <6 years and a semi-structured diagnostic interview (KSADS) for children > 6 years, and determined the need to receive drug treatment for each child according to the type of diagnosis, severity of symptoms, effect of behavior problems on children’s performance, and non-responsiveness to behavior interventions.

The Kiddie-schedule for affective disorders and schizophrenia-present and lifetime (KSADS) is a semistructured diagnostic interview that can be performed by a trained specialist for children aged 6–18 years. The interview consists of a screening interview, which evaluates symptoms of mood disorders, psychosis, anxiety disorders, elimination disorders (enuresis and encopresis), attention-deficit/hyperactivity disorder (ADHD), and oppositional defiant disorder, tick, and alcohol substance abuse. It also has five supplementary appendices in domains, including mood, anxiety, behavioral disorders, psychotic disorders, and substance abuse. The appendices will be completed if problematic domains are found during the screening interview. Hence, the interview provides the possibility of diagnosis of DSM-IV diagnoses in a wide range of psychiatric disorders. Interrater agreement in scoring screens and diagnoses was reported high (93–100%). 

Test-retest reliability kappa coefficient were in the excellent range for present and/or lifetime diagnoses of major depression, any bipolar, generalized anxiety, conduct, and oppositional defiant disorder (0.77–1.00) and in the good range concerning present diagnoses of posttraumatic stress disorder and attention-deficit hyperactivity disorder (0.63–0.67). The existing data support the validity of KSADS diagnoses (23,24). Intelligence status of the patient was assessed according to parental reports of childhood developmental history and patient current cognitive performance, and by the Wechsler Intelligence Test at ages over 5 years, and intellectual disability was being diagnosed according to DSM-IV criteria. After a written consent was obtained from the parents, the patients were randomly divided into 2 groups: the first group underwent treatment with a risperidone tablet in a dose range from 0.75 mg/d to 1.5 mg/d, based on patient’s weight. Therefore, for children weighing less than 50 kg, risperidone was started at a dose of 0.25 mg/d and gradually increased to the final dose of 0.75 mg/d over a period of one week. Whereas, for children more than 50 kg, the starting dose of risperidone was 0.5 mg/d and the final dose was 1.5 mg/d. In the second group, for children less than 50 kg, buspirone was started at a dose of 2.5 mg/d, and was raised to 15 mg/d over two weeks, and in children more than 50 kg, the initial dose was 5 mg/d and the final dose was 15 mg/d. The treatment was continued for 8 weeks in both groups. After completing the 8 week treatment period, the dosage of the first drug was reduced and discontinued and then, patients in the first and second groups were given, respectively, buspirone and risperidone with the mentioned conditions. The second treatment period was continued for 8 weeks. Some patients were already under treatment with risperidone, the dose was only adjusted if needed, and they were placed in the buspirone group after 8 weeks.

Considering that all patients were in the intellectual disability range upon the initial assessment, they were evaluated in terms of changes in the severity of behavioral disorders in three stages of before treatment, after treatment with risperidone, and after treatment with buspirone by the NCBRF, which was completed by parents.

NCBRF has been designed specifically for the assessment of behavioral problems in children with intellectual disability. It consists of 10 items on social competence and 66 items on behavioral problems that comprise 6 subscales, including conduct problems, insecure/anxious, hyperactive, self-injury/stereotypic self-isolated/ritualistic, and overly sensitive (25-27). 

Overall improvement was measured by the 7-point CGI after the end of the 8-week treatment period through asking question from parents and assessment of children by the therapist (28,29). This scale has been used to assess disease severity and overall improvement, and is scored on a 1–7 scale; the lower scores indicate the decrease of psychopathology and more therapeutic effects. The phenylalanine level of patients was measured at least one time during treatment with each drug. The exclusion criteria were an increase of behavioral disorders, adverse effects from the drugs, existence of a severe chronic diseases, and phenylalanine more than 10 mg/dl during the treatment period. The data obtained from NCBRF and CGI together with other PKU related data were collected from the dossiers of patients and were recorded on data collection forms. The data were analyzed using SPSS software (ver. 16).

## Results

From 42 patients with PKU patients covered by the PKU Clinic of the Besat Hospital of Hamadan, 26 cases who had severe behavior problems were referred to a pediatric psychiatrist, from them, 22 cases were eligible to enter the project. Twenty eligible children were treated with risperidone and buspirone. Four cases withdrew from the project during the treatment and 3 cases became restless during the discontinuation of risperidone and starting buspirone. Therefore, risperidone was again started for them. 

Finally, 13 patients were able to complete the 16-week period of treatment with the two drugs. The mean age of these patients was 7.71 ± 4.18. Eight of them were boys and five were girls. All 13 cases were diagnosed late and none of the patients had malignant PKU. 

None of the patients had phenylalanine more than 10 mg/ dl during treatment, and the mean serum phenylalanine of the patients was not significantly different during treatment with the two drugs (p = 0.537).

The most common psychiatric diagnosis in 22 eligible patients was an intellectual disability accompanied by pervasive developmental disorders (not otherwise specified) (PDD NOS) and intellectual disability accompanied by autistic disorder (Table 1).

Assessment of the severity of behavioral disorder, before and after treatment with risperidone by NCBRF showed significant decreases in the severity of subscales of hyperactivity (p=0.001), disruptive/ stereotypic (p=0.009), and conduct problems (p=0.043). 

Other aspects of behavior problems had no significant differences (Table 2). 

In comparison of the effects of risperidone and buspirone, a decrease in the subscales of hyperactivity (p=0.024) and disruptive/stereotypic (p=0.042) in the risperidone group was significantly different from the buspirone group. Also, a decrease in the subscales of self-isolation (p=0.004) and insecure/anxious (p=0.031) in risperidone group was significantly different from the buspirone group; however, the comparison of before and after treatment was not significantly different (Table 2). 

Concerning overall clinical efficacy according to CGI, risperidone (2.61 ± 0.50) was significantly more effective compared to buspirone (4.23 ± 1.48; p=0.006).

## Discussion

The findings of our study reveal that risperidone is effective in the control of hyperactivity, disruptive/ stereotypic behaviors, and conduct problems in children with PKU .This finding is in accordance with other studies showing the effectiveness of risperidone in the decrease of hyperactivity, disruptive/stereotypic behaviors, and conduct problems in children with autistic disorder and intellectual disability (5-18, 30). Risperidone showed no significant differences in the control of other subscales of NCBRF (insecure/anxious, self-isolation, and overly sensitive), which may be justified by the type of disease in the studied group.

**Table 1:** Psychiatric Diagnosis in Eligible PKU Patients

**Percent**	**number**	**Diagnosis**
18.18%	4	ID
27.27%	6	ID-PDD NOS
27.27%	6	ID-Autistic disorder
4.54%	1	ID-ADHD
18.18%	4	ID-ADHD-ODD
4.54%	1	ADHD-ODD
	22	Total

ID: Intellectual Disability; PDD N.O.S: Pervasive Developmental Disorder not otherwise specified; ADHD: Attention Deficit hyperactivity Disorder; ODD:Oppositional-Defiant Disorder

**Table 2:** The Severity of Behavior Disorder in PKU Patients Before and After Treatment with Risperidone and Buspirone According to Nisonger Child Behavior Rating Form

Risperidone vs Buspirone	after treatment with Buspirone			after treatment with Risperidone			Pre-treatment		
P-value	P-value	SD	mean	P-value	SD	mean	SD	mean	
1.000	0.159	4.78	7.76	0.099	3.91	7.76	3.77	5.69	Compliant/Calm
0.927	0.064	3.04	5.46	0.054	3.23	5.53	3.06	3.61	Adaptive Social
0.111	0.744	8.96	17.15	0.043	4.46	13.61	8.83	18.15	Conduct Problem
0.031	0.759	4.61	7.46	0.153	3.66	5.61	4.95	7.92	Insecure/Anxious
0.024	0.045	3.51	15.00	0.001	2.75	11.46	5.18	18.53	Hyperactivity
0.042	0.153	4.80	5.07	0.009	2.85	2.84	4.93	6.69	Self-Injury/ Stereotypic
0.004	0.455	2.58	6.00	0.066	2.47	4.53	5.45	6.92	Self-Isolation
0.551	0.481	3.37	8.30	0.072	3.22	7.69	3.68	9.07	Overly Sensitive

Buspirone was only effective in the control of hyperactivity; however, risperidone was significantly more effective. This finding is consistent with the results of Gualtieri and Stanislav et al studies (19,20).

The notable point is the ineffectiveness of buspirone in reducing anxiety symptoms and risperidone is more effective in controlling anxiety symptoms. Considering that buspirone is an anti-anxiety drug, this finding seems contradictory. Nevertheless, given that the majority of children in this study had comorbidity with the range of pervasive developmental disorder; therefore, the symptoms reported by parents about their children’s anxiety in some items of NCBRF can result from social interaction disorder and its caused self-isolation, but is not due to social anxiety. Some studies have revealed that risperidone decreases hyperactivity, irritability, and stereotypic behaviors in autistic disorder and also increases verbal communication that can result in the reduction of parental’ reports of anxiety behavior. 

**In conclusion, **this study shows that in spite of more advantages of risperidone than buspirone, in cases where the risperidone is not effective, buspirone could be administered as an alternative. 

Studies with a larger sample size and on other groups with primary brain disorders are suggested.

## References

[1] Smith I, Nowles JK (2000). Behaviour in early treated phenylketonuria: a systematic review. Eur J Pediatr.

[2] Targum SD, Lang W (2009). Neurobehavioral Problems Associated with Phenylketonuria. Psychiatry (Edgemont).

[3] Luciana M, Hanson K L, Whitley C B (2004). A preliminary report on dopamine system reactivity in PKU: acute effects of haloperidol on neuropsychological, physiological, and neuroendocrine functions. Psychopharmacology.

[4] Pappadopulos E, Woolston S, Chait A, Perkins M, Connor DF, Jensen P S (2006). Pharmacotherapy of Aggression in Children and Adolescents: Efficacy and Effect Size. J CDN ACAD Child Adolesc Psychiatry.

[5] Shea S, Turgay A, Carroll A, Schulz M, Orlik H, Smith I (2004). Risperidone in the Treatment of Disruptive Behavioral Symptoms in Children With Autistic and Other Pervasive Developmental Disorders. Pediatrcs.

[6] Miral S, Gencer O, Inal-Emiroglu FN, Baykara B, Baykara A, Dirik E (2008). Risperidone versus haloperidol in children and adolescents with AD: a randomized, controlled, doubleblind trial. Eur Child Adolesc Psychiatry.

[7] Aman MG, Hollway JA, McDougle CJ, Scahill L, Tierney E, McCracken JT (2008). Cognitive effects of risperidone in children with autism and irritable behavior. J Child Adolesc Psychopharmacol.

[8] Pandina GJ, Bossie CA, Youssef E, Zhu Y, Dunbar F (2007). Risperidone improves behavioral symptoms in children with autism in a randomized, double-blind, placebocontrolled trial. J Autism Dev Disord.

[9] Luby J, Mrakotsky C, Stalets MM, Belden A, Heffelfinger A, Williams M (2006). Risperidone in preschool children with autistic spectrum disorders: an investigation of safety and efficacy. J Child Adolesc Psychopharmacol.

[10] Nagaraj R, Singhi P, Malhi P (2006). Risperidone in children with autism: randomized, placebo controlled, double blind study. J Child Neurol.

[11] Haas M, Karcher K, Pandina GJ (2008). Treating Disruptive Behavior Disorders with Risperidone: A 1-Year, Open-Label Safety Study in Children and Adolescents. Journal of Child and Adolescent Psychopharmacology.

[12] Jensen P, Buitelaar J, Pandina G, Binder C, Haas M (2007). Management of psychiatric disorders in children and adolescents with atypical antipsychotics. Eur Child Adolesc Psychiatry.

[13] Pandina G, Aman M, Findling R (2006). Risperidone in the management of disruptive behavior disorders. J Child Adolesc Psychopharmacol.

[14] Reyes M, Buitelaar J, Toren P, Augustyns I, Eerdekens M (2006). A randomized, double-blind, placebo-controlled study of risperidone maintenance treatment in children and adolescents with disruptive behavior disorders. Am J Psychiatry.

[15] Reyes M, Croonenberghs J, Augustyns I, Eerdekens M (2006). Long-term use of risperidone in children with disruptive behavior disorders and subaverage intelligence: Efficacy, safety, and tolerability. J Child Adolesc Psychopharmacol.

[16] Croonenberghs J, Fegert JM, Findling RL, De Smedt G, Van Dongen S (2005). Risperidone Disruptive Behavior Study Group: Risperidone in children with disruptive behavior disorders and subaverage intelligence: A 1-year, openlabel study of 504 patients. J Am Acad Child Adolesc Psychiatry.

[17] Aman M G, Smedt G D, Derivan A, Lyons B, Findling R L (2002). Double-Blind, Placebo-Controlled Study of Risperidone for the Treatment of Disruptive Behaviors in Children With Subaverage Intelligence. Am J Psychiatry.

[18] Snyder R, Turgay A, Aman M, Binder C, Fisman S, Carroll A (2002). Risperidone Conduct Study Group: Effects of risperidone on conduct and disruptive behavior disorders in children with subaverage IQs. J Am Acad Child Adolesc Psychiatry.

[19] Gualtieri T (1991). Buspirone for the Behavior problems of patients with Organic Brain Disorders. J Clin Psycopharmacol.

[20] Stanislav S, Fabre T, Crismon L, Childs A (1994). Buspirone’s Efficacy in Organic-Induced Aggression. J Clin Psycopharmacol.

[21] Realmuto GM, August GJ, Garfinkel BD (1989). Clinical effect of buspirone in autistic children. J Clin Psychopharmacol.

[22] Ratey J, Sovner R, Parks A, Rogentine K (1991). Buspirone treatment of aggression and anxiety in mentally retarded patients: a multiple-baseline, placebo lead-in study. J Clin Psychiatry.

[23] Sorensen MJ, Thomsen PH, Bilenberg N (2007). Parent and child acceptability and staff evaluation of K-SADA-PL: a pilot Study. European Child and Adolescent Psychiatry.

[24] Kaufman J, Brimaher B, Bren, D, Rao U, Flynn C, Moreci P (1997). Schedule for affective disorders and Schizophrenia For school –age children – present and lifetime version- (K-SADS-PL): initial reliability and validity date. Journal of the American Academy of Child and Adolescent Psychiatry.

[25] Aman MG, Tassé MJ, Rojahn J, Hammer D (1996). The Nisonger CBRF: a child behavior rating form for children with developmental disabilities. Research in Developmental Disabilities.

[26] Tassé MJ, Aman MG, Hammer D, Rojahn J (1996). The Nisonger Child Behavior Rating Form: age and gender effect and norms. Research in Developmental Disabilities.

[27] Norris M, Lecavalier L (2011). Evaluating the validity of the Nisonger Child Behavior Rating Form – Parent Version. Research in Developmental Disabilities.

[28] Guy W, Rockville MD (1976). Clinical global impressions. ECDEU Assessment Manual for Psychopharmacology.

[29] National Institute of Mental Health (1985). CGI (Clinic Global Impression) Scale. Psychopharmacology Bull.

[30] Marder S, Hurford IM, van Kammen DP, Sadock BJ, Sadock VA, Pedro R ( 2009). Second- Generation Antipsychotics: Biological Therapies. Kaplan & Sadock’s Comprehensive Textbook of Psychiatry.

